# Influence of Anxiety Level and Degree of Alexithymia on Quality of Life in Adult Patients With Primary Immune Thrombocytopenia

**DOI:** 10.62641/aep.v53i6.2026

**Published:** 2025-12-17

**Authors:** Shunyu Liang, Tao Wu, Liping Chai, Tao Ai, Shan Lu

**Affiliations:** ^1^Department of Hematology, The 940th Hospital of Joint Logistics Support Force of Chinese People’s Liberation Army, 730050 Lanzhou, Gansu, China; ^2^Department of Health, The 940th Hospital of Joint Logistics Support Force of Chinese People’s Liberation Army, 730050 Lanzhou, Gansu, China; ^3^Department of Obstetrics, The 940th Hospital of Joint Logistics Support Force of Chinese People’s Liberation Army, 730050 Lanzhou, Gansu, China

**Keywords:** thrombocytopenia, anxiety, alexithymia, quality of life

## Abstract

**Background:**

Primary immune Thrombocytopenia (ITP) is an autoimmune disease characterised by Thrombocytopenia, which can cause physical symptoms such as bleeding and impose a heavy burden on patients’ mental health and quality of life (QOL). This study aims to investigate the impact of anxiety level and degree of alexithymia on the QOL of adult patients with ITP.

**Methods:**

This investigative study included 148 patients with ITP attending our haematology department from June 2021 to June 2023. The following scales were used: the Hamilton Anxiety Scale (HAMA) to assess the patients’ anxiety level, the Toronto Alexithymia Scale-20 (TAS-20) to assess the degree of alexithymia and the ITP-Patient Assessment Questionnaire (ITP-PAQ) to assess QOL. Pearson correlation and multifactor linear regression analyses were used to explore the relationship among anxiety level, degree of dysphoria and QOL.

**Results:**

The mean HAMA score of 148 patients with ITP was 14.31 ± 3.61, of which 146 had varying degrees of anxiety. The mean TAS-20 score was 56.11 ± 8.41, and 106 cases had varying degrees of alexithymia. Pearson correlation analysis showed that HAMA scores (r = –0.316, *p* < 0.001) and TAS-20 scores (r = –0.254, *p* = 0.002) were significantly negatively correlated with ITP-PAQ scores. The results of multifactorial linear regression analysis showed that anxiety level (*p* < 0.001), alexithymia (*p* = 0.015), diabetes mellitus comorbidities (*p* = 0.046), stage of disease (*p* = 0.027) and platelet (PLT) level (*p* = 0.032) were independent risk factors for the QOL of patients with ITP.

**Conclusion:**

Anxiety level and alexithymia degree significantly affect the QOL of patients with ITP and are independent risk factors for QOL. Clinical work should pay attention to the psychological state of patients with ITP and the timely identification and intervention for anxiety and alexithymia to improve QOL.

## Introduction

Primary immune Thrombocytopenia (ITP) is an autoimmune disease characterised by Thrombocytopenia, the pathogenesis of which involves abnormal destruction and insufficient production of platelets by the immune system [1]. The annual incidence of ITP in adults varies geographically and by age. Recent population-based studies from Europe have reported overall incidence rates ranging from 1.6 to 3.9 per 100,000 adults per year, with higher rates observed in the elderly and a slight female predominance [2, 3]. In contrast to other systemic diseases, ITP presents unique characteristics that distinguish its impact on psychological status and quality of life (QOL); it is dominated by bleeding risks (e.g. skin/mucous membrane bleeding and even life-threatening intracranial bleeding in severe cases), which are acute, unpredictable and directly linked to platelet count fluctuations [4]. While many systemic diseases may affect mental health, ITP-specific stressors include the constant fear of sudden bleeding episodes, activity restrictions to avoid trauma and anxiety about treatment response variability; these factors create a distinct chronic stress model. Although most patients can be relieved after active treatment, recurrent attacks and treatment-related adverse effects exacerbate this unique burden [5]. The uncertainty of bleeding events places patients with ITP in a state of persistent hypervigilance, which differs from the psychological impact of diseases with more predictable progression or symptom patterns. Psychological problems of varying degrees are common in patients with chronic diseases [6]. The psychological state of ITP patients, as a member of the chronic disease group, deserves equal attention.

Anxiety, a common negative emotion, is more prevalent in patients with ITP [7]. Anxiety influences not only the patient’s psychological state but also the immune system through neuroendocrine and other mechanisms, thereby interfering with the therapeutic effects of the disease and the recovery process [8]. Anxiety may lead to increased levels of pro-inflammatory cytokines in the body, which may further exacerbate immune disorders and platelet damage in patients with ITP [9]. Alexithymia is a personality trait characterised by difficulty recognising and describing one’s emotions, lack of fantasy and extroverted thinking [10]. Current research has focused on the presence of alexithymia in patients with chronic diseases and their impact on disease management and patients’ lives [11]. QOL, as an important indicator to evaluate the health status of patients with chronic diseases, covers multiple dimensions such as physiological, psychological and social functions [12]. The decline in the QOL of patients with ITP is related not only to the symptoms and severity of the disease itself but also to psychological factors [13]. The QOL of patients with ITP is significantly lower than that of healthy people, and this impact is widely related to work, study, daily affairs, physical fitness, athletic ability and sex life [14]. Prolonged anxiety may lead to sleep disorders and loss of appetite, which in turn affects the physiological functions of the body and reduces the QOL [15]. Alexithymia also has a negative impact on QOL because it prevents patients from dealing with their emotions, which can lead to strained interpersonal relationships and reduced social support [16].

The treatment of patients with ITP has continuously improved and includes pharmacological treatments such as glucocorticoids, immunoglobulins and thrombopoietin receptor agonists as well as surgical treatments such as splenectomy; however, limited information is available about the psychological state and its impact on the QOL of the patients [1, 17]. Although prior evidence points to heightened anxiety and alexithymia in adults with ITP, the extent to which these psychological dimensions concurrently and independently shape health-related QOL has not yet been systematically quantified in this population. An in-depth exploration of the intrinsic links between the three may not only contribute to a comprehensive understanding of the burden of disease in patients with ITP but may also provide new directions for clinical interventions. This study aimed to systematically assess the level of anxiety and the degree of alexithymia in adult patients with ITP and to explore its impact on QOL. Results will provide clinicians with a comprehensive perspective on patient management, helping them to focus on patients’ physical symptoms as well as their mental health and QOL, to provide effective healthcare.

## Materials and Methods

### Patient Population

This investigational study included 148 patients with ITP who were treated between June 2021 and June 2023 in the 940th Hospital of Joint Logistics Support force of Chinese People’s Liberation Army. The inclusion criteria were as follows: (1) patients aged ≥18 years; (2) patients who met the Chinese Guidelines for the Diagnosis and Treatment of ITP in Adults (2020 edition): (a) at least two consecutive routine blood tests suggesting a reduced platelet count, with no obvious abnormalities in the morphology of blood cells on microscopic examination of peripheral blood smears; (b) the spleen is generally not enlarged; (c) bone marrow cell morphology characterised by increased or normal megakaryocytes with impaired maturation; (d) rule out other secondary thrombocytopenia [18]; (3) patients who were clearly conscious, possessed basic comprehension and communication skills, and were able to cooperate in completing the questionnaires; and (4) patients with complete clinical data. The exclusion criteria were as follows: (1) patients with comorbidities of other haematological disorders (e.g. leukaemia, aplastic anaemia) or malignant tumours; (2) patients with comorbidities of severe dysfunctions of vital organs such as the heart, liver, kidney, etc.; (3) patients who had received medication (e.g. antipsychotics, high-dose glucocorticosteroids, etc.) or psychological interventions that might affect their psychological state within 3 months; (4) patients with severe mental illness or cognitive impairment, and patients who were unable to complete the questionnaire. All patients voluntarily signed an informed consent form. The study complied with the guidelines of the Declaration of Helsinki and was approved by the Ethics Committee of the 940th Hospital of the Joint Logistic Support Force of the People’s Liberation Army of China (Ethical Approval Number: 2024KYLL047).

### Data Collection

General and clinical data of patients were collected through the hospital’s electronic medical record system. General information included age, sex, smoking (1: yes; 0: no), alcohol consumption (1: yes; 0: no), hypertension (1: yes; 0: no), diabetes (1: yes; 0: no) and coronary heart disease (1: yes; 0: no). Clinical data included disease stage [4] (1: initial ITP [<3 months after diagnosis]; 0: persistent or chronic ITP [≥3 months after diagnosis]), treatment modalities (1: second-line; 0: first-line), platelet (PLT) levels (1: ≤100 × 10^9^/L; 0: >100 × 10^9^/L), antinuclear antibodies (1: positive; 0: negative) and *Helicobacter pylori* (Hp) infection (1: positive; 0: negative). In addition, anxiety level, alexithymia level and QOL were assessed using appropriate scales.

### Survey Instrument

The Hamilton Anxiety Scale (HAMA) is a classic scale used to assess the severity of anxiety symptoms and was developed by Hamilton in 1959 [19]. The scale contains 14 items, covering anxious mood, tension, fear, insomnia and other aspects. Each item is scored on a 5-point scale (0–4) based on the severity of symptoms, with 0 being asymptomatic and 4 being extremely severe. Scores on the HAMA scale range from 0 to 56, with higher scores indicating more severe anxiety. The HAMA scale has been proved by Chinese scholars to have high reliability and validity [20]. In this study, the Cronbach’s alpha coefficient of the HAMA scale was 0.83. The Toronto Alexithymia Scale-20 (TAS-20) is a scale specifically designed to assess the degree to which an individual has difficulty describing emotions [21]. The scale consists of 20 items divided into three factors: difficulty identifying feelings, difficulty describing feelings and externally oriented thinking. Each item is rated on a 5-point scale from 1 to 5, with higher total scores indicating greater levels of dysphoria. The three commonly used thresholds for TAS-20 scores are as follows: no alexithymia: 20–50; low alexithymia: 51–60; and high Alexithymia: 61–100 [22]. TAS-20 has been proved to have good reliability and validity in China [23]. In this study, the Cronbach’s alpha coefficient of the TAS-20 scale was 0.86. The ITP-Patient Assessment Questionnaire (ITP-PAQ) is an assessment tool specifically designed to evaluate the QOL of patients with ITP, and it contains a multidimensional evaluation of 10 subscales [24]. ITP-PAQ consists of 10 subscales with a total of 44 questions. We divided the 10 subscales into three modules: the emotional module (anxiety, psychology, fear), the physical module (symptoms, fatigue/sleep, exercise) and the other module (work, social interaction, women, overall QOL). Each item in the ITP-PAQ questionnaire is scored using the Likert scale. Both 5-point and 7-point scales exist simultaneously, so the score of each item needs to be converted to 0–100 points during statistical analysis (linear transformation). The transformed score was calculated using the equation: [(raw score – 1)/(maximum raw score – 1)] × 100. The module score is the average of its constituent subscales, and the ITP-PAQ total score is the average of all 10 subscales. Higher ITP-PAQ scores indicate higher QOL. ITP-PAQ has good reliability and validity [25]. In this study, the Cronbach’s alpha coefficient of the ITP-PAQ scale was 0.82.

### Questionnaire Quality Control

Prior to the survey, uniform training was provided to participating healthcare professionals and research assistants, covering the theoretical basis of each scale, scoring criteria, guideline specifications and communication skills to avoid human-led bias. The questionnaires were filled out in a one-to-one mode in a quiet and private environment, with the investigator explaining the purpose of the study and the patients filling out and submitting the questionnaires on the spot. For patients with poor vision or low literacy, the investigator read the questions aloud in a neutral tone and assisted in recording to ensure independent judgement. A total of 160 questionnaires were distributed, and 148 were effectively retrieved, with a recovery rate of 92.5%. At the data entry stage, we used double entry and logical checks, where two independent data entry clerks entered data separately and cross-checked them to detect and correct entry errors. Data were automatically checked using logical rules to ensure effective control of data ranges, missing values and outliers.

### Statistical Analysis

SPSS 26.0 (IBM Corp., Armonk, NY, USA) statistical software was used to analyse the collected data. Before performing statistical analysis, we conducted normality and homogeneity of variance tests on all measurement data. The Kolmogorov-Smirnov method was used for normality testing, and the Levene method was used for homogeneity of variance testing. All variables passed the normality and homogeneity of variance tests. Measurement data were expressed as mean ± standard deviation ($\bar{x}$ ± s). Independent sample *t* test was used for comparison between two groups, and ANOVA was used for comparison among multiple groups. Count data were expressed as frequency and percentage (%), and the χ^2^ test was used for comparison between groups. Pearson correlation analysis was used to explore the correlation among anxiety level, alexithymia degree, QOL and scores of each dimension. Correlation coefficient (r) and its 95% confidence interval (CI) were calculated. Through multiple linear regression analysis, with the total score of QOL as the dependent variable and statistically significant variables in the univariate analysis as the independent variables, a step-by-step regression model was constructed to explore the independent risk factors affecting the QOL of patients with ITP. In the stepwise multiple linear regression, all categorical predictors were converted into binary dummy variables: Hp infection (Hp-positive = 1, Hp-negative = 0), diabetes mellitus (yes = 1, no = 0), disease stage (initial ITP = 1, persistent/chronic ITP = 0) and platelet level (≤100 × 10^9^/L = 1, >100 × 10^9^/L = 0). The significance level (*p* value) for entering the model was set at 0.05, and the significance level (*p* value) for excluding the model was set at 0.10. Through this criterion, variables that have a significant independent impact on the dependent variable were screened out. The coefficient of determination (R^2^) was calculated to analyse the extent to which the model explained the dependent variable. The F-test was used to assess the significance of the model. For collinearity diagnosis by variance inflation factor (VIF), VIF <5 indicates the lack of a collinearity problem between variables. *p* < 0.05 was considered to be statistically significant.

## Results

### Overall QOL Scores of Patients

The mean ITP-PAQ score for all patients was 68.97 ± 9.86. The ITP-PAQ score of patients with age >50 years was significantly lower than that of patients with age ≤50 years (*p* = 0.018). Patients with comorbid diabetes mellitus, initial patients, patients with PLT ≤100 × 10^9^/L and patients with comorbid Hp infection had lower ITP-PAQ scores than patients in the same category (*p* < 0.05, Table 1).

**Table 1.** **Overall ITP-PAQ scores of patients**.

Variables		*n* (%)	ITP-PAQ, Mean ± SD	Statistic	*p*
Total		148 (100)	68.97 ± 9.86		
Age				t = 2.39	0.018
	≤50	82 (55.41)	70.67 ± 9.55		
	>50	66 (44.59)	66.84 ± 9.90		
Gender				t = 0.47	0.640
	Female	88 (59.46)	69.28 ± 9.13		
	Male	60 (40.54)	68.51 ± 10.91		
Smoking				t = 0.99	0.322
	No	114 (77.03)	69.41 ± 9.61		
	Yes	34 (22.97)	67.49 ± 10.67		
Drinking				t = 0.06	0.952
	No	122 (82.43)	68.99 ± 9.98		
	Yes	26 (17.57)	68.86 ± 9.49		
Hypertension				t = 0.70	0.484
	No	117 (79.05)	69.26 ± 9.76		
	Yes	31 (20.95)	67.86 ± 10.33		
Diabetes				t = 2.71	0.008
	No	124 (83.78)	69.91 ± 9.00		
	Yes	24 (16.22)	64.08 ± 12.61		
Coronary heart disease				t = –1.18	0.242
	No	131 (88.51)	68.62 ± 9.71		
	Yes	17 (11.49)	71.61 ± 10.89		
Disease stage				t = 2.60	0.010
	Persistent or chronic ITP	89 (60.14)	70.65 ± 10.24		
	Initial ITP	59 (39.86)	66.43 ± 8.74		
Treatment				t = –0.88	0.381
	First-line	77 (52.03)	68.28 ± 10.34		
	Second-line	71 (47.97)	69.71 ± 9.32		
PLT level				t = 2.29	0.024
	>100 × 10^9^/L	73 (49.32)	70.82 ± 9.66		
	≤100 × 10^9^/L	75 (50.68)	67.16 ± 9.78		
Antinuclear antibody				t = 0.30	0.767
	Negative	113 (76.35)	69.10 ± 10.49		
	Positive	35 (23.65)	68.53 ± 7.57		
Hp infection				t = 2.59	0.011
	Negative	91 (61.49)	70.60 ± 10.00		
	Positive	57 (38.51)	66.36 ± 9.12		

ITP, Primary immune Thrombocytopenia; ITP-PAQ, Primary immune Thrombocytopenia-Patient Assessment Questionnaire; SD, standard deviation; PLT, platelet; Hp, Helicobacter pylori.

### QOL Scores in Different Dimensions

In the emotional scoring module of the ITP-PAQ scale, patients with age >50 years (t = 2.19, *p* = 0.030) and who had combined diabetes (t = 2.89, *p* = 0.005), initial patients (t = 2.81, *p* = 0.006) and combined Hp infection (t = 2.65, *p* = 0.009) had lower scores than patients in the same category (Table 2). In the physical scoring module, lower scores were observed in patients with combined diabetes (t = 2.46, *p* = 0.015), initial patients (t = 2.17, *p* = 0.032), patients with PLT ≤100 × 10^9^/L (t = 2.56, *p* = 0.011) and patients with Hp infection (t = 3.06, *p* = 0.003). In other scoring modules, lower scores were observed in patients with age >50 (t = 2.73, *p* = 0.007), initial patients (t = 2.57, *p* = 0.011) and patients with PLT ≤100 × 10^9^/L (t = 1.98, *p* = 0.049).

**Table 2.** **ITP-PAQ scores of patients divided according to different modules**.

Variables		*n* (%)	Emotion module, Mean ± SD	Physical module, Mean ± SD	Other modules, Mean ± SD
Total		148 (100)	67.89 ± 9.52	70.10 ± 11.67	68.91 ± 10.09
Age					
	≤50	82 (55.41)	69.40 ± 9.20*	71.72 ± 11.46	70.90 ± 9.83**
	>50	66 (44.59)	66.00 ± 9.65	68.09 ± 11.70	66.44 ± 9.94
Gender					
	Female	88 (59.46)	68.14 ± 9.00	70.64 ± 10.66	69.07 ± 9.40
	Male	60 (40.54)	67.52 ± 10.31	69.32 ± 13.06	68.68 ± 11.11
Smoking					
	No	114 (77.03)	68.32 ± 9.21	70.40 ± 11.34	69.49 ± 9.94
	Yes	34 (22.97)	66.41 ± 10.51	69.09 ± 12.84	66.97 ± 10.51
Drinking					
	No	122 (82.43)	67.89 ± 9.58	69.98 ± 11.86	69.09 ± 10.16
	Yes	26 (17.57)	67.85 ± 9.44	70.65 ± 10.94	68.08 ± 9.94
Hypertension					
	No	117 (79.05)	68.27 ± 9.61	70.27 ± 11.28	69.23 ± 10.03
	Yes	31 (20.95)	66.42 ± 9.19	69.45 ± 13.21	67.71 ± 10.42
Diabetes					
	No	124 (83.78)	68.85 ± 8.72**	71.12 ± 10.83*	69.76 ± 9.31
	Yes	24 (16.22)	62.88 ± 11.88	64.83 ± 14.46	64.54 ± 12.81
Coronary heart disease					
	No	131 (88.51)	67.53 ± 9.41	69.77 ± 11.49	68.57 ± 10.01
	Yes	17 (11.49)	70.65 ± 10.22	72.65 ± 13.10	71.53 ± 10.68
Disease stage					
	Persistent or chronic ITP	89 (60.14)	69.55 ± 10.21**	71.78 ± 11.75*	70.62 ± 10.37*
	Initial ITP	59 (39.86)	65.37 ± 7.80	67.58 ± 11.18	66.34 ± 9.16
Treatment					
	First-line	77 (52.03)	67.12 ± 10.08	69.34 ± 12.16	68.39 ± 10.38
	Second-line	71 (47.97)	68.72 ± 8.88	70.93 ± 11.14	69.48 ± 9.81
PLT level					
	>100 × 10^9^/L	73 (49.32)	69.34 ± 9.71	72.55 ± 11.21*	70.56 ± 9.94*
	≤100 × 10^9^/L	75 (50.68)	66.47 ± 9.17	67.72 ± 11.69	67.31 ± 10.05
Antinuclear antibody					
	Negative	113 (76.35)	67.89 ± 10.07	70.41 ± 12.36	69.00 ± 10.60
	Positive	35 (23.65)	67.86 ± 7.60	69.11 ± 9.17	68.63 ± 8.37
Hp infection					
	Negative	91 (61.49)	69.49 ± 9.36**	72.36 ± 11.92**	69.93 ± 10.38
	Positive	57 (38.51)	65.32 ± 9.28	66.49 ± 10.37	67.28 ± 9.47

*Compared with another subgroup, independent sample *t*-test, *p* < 0.05. **Compared with another subgroup, independent sample *t*-test, *p* < 0.01. ITP, Primary immune Thrombocytopenia; ITP-PAQ, Primary immune Thrombocytopenia-Patient Assessment Questionnaire; SD, standard deviation; PLT, platelet; Hp, Helicobacter pylori.

### Levels of Anxiety and Alexithymia

The average HAMA score of all patients was 14.31 ± 3.61, among which 146 patients showed different degrees of anxiety (Table 3). Sixty-one cases may have anxiety, 77 cases definitely have anxiety, and 8 cases must have significant anxiety. The mean TAS-20 score for all patients was 56.11 ± 8.41, and 106 patients showed varying degrees of alexithymia. A total of 59 and 47 patients had low and high levels of alexithymia, respectively (Table 4).

**Table 3.** **Level of anxiety**.

Variables	Score	Negative	May have anxiety	Definitely have anxiety	Significant anxiety
Anxiety^a^	14.31 ± 3.61	2	61	77	8

^a^HAMA score: <7 No symptoms of anxiety; 7–14 May have anxiety; 14–21 Definitely have anxiety; ≥21 must have significant anxiety; HAMA, Hamilton Anxiety Scale.

**Table 4.** **Level of alexithymia**.

Variables	Score	No Alexithymia	Low Alexithymia	High Alexithymia
Alexithymia^a^	56.11 ± 8.41	42	59	47

^a^Alexithymia: No Alexithymia: 20–50 scores; Low Alexithymia: 51–60 scores; High Alexithymia: 61–100 scores.

### Correlation Analysis of Anxiety, Alexithymia and Overall QOL

**Fig. 1.**
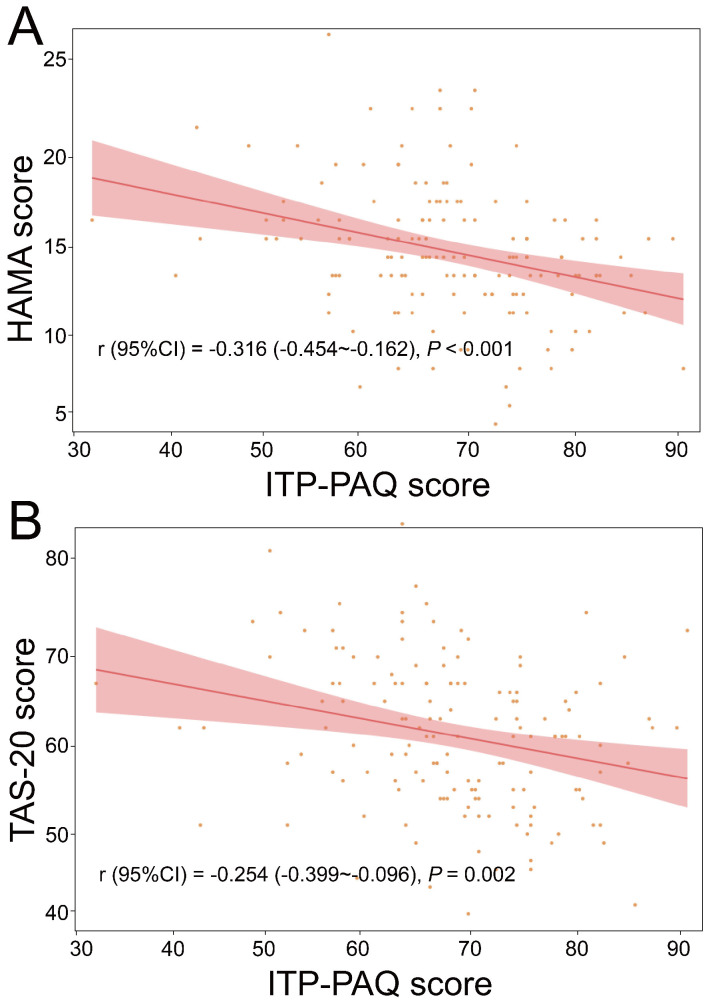
**Correlation analysis of ITP-PAQ with HAMA and TAS-20**. (A) HAMA; (B) TAS-20. TAS-20, Toronto Alexithymia Scale-20; ITP-PAQ, Primary immune Thrombocytopenia-Patient Assessment Questionnaire; HAMA, Hamilton Anxiety Scale.

The results of the Pearson correlation analysis showed that HAMA scores (r = –0.316, 95% CI: –0.454~–0.162, *p* < 0.001) as well as TAS-20 scores (r = –0.254, 95% CI: –0.399~–0.096, *p* = 0.002) were significantly negatively correlated with ITP-PAQ scores (Fig. 1).

### Correlation Between Anxiety, Alexithymia and QOL in Different Modules

**Fig. 2.**
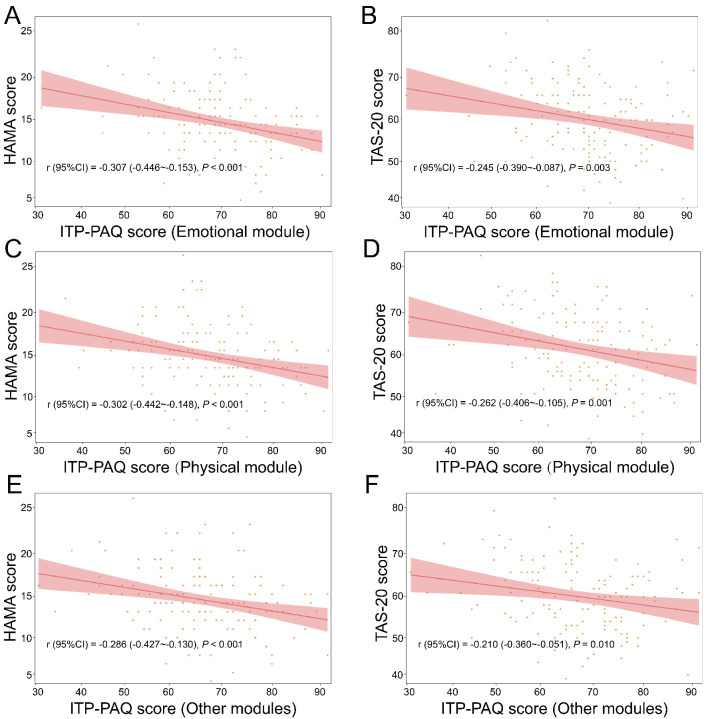
**Correlation analysis of different modules of ITP-PAQ with HAMA and TAS-20**. (A,B) Emotional module; (C,D) Physical module; and (E,F) Other modules. ITP-PAQ, Primary immune Thrombocytopenia-Patient Assessment Questionnaire; TAS-20, Toronto Alexithymia Scale-20; HAMA, Hamilton Anxiety Scale.

The HAMA score (r = –0.307, 95% CI: –0.446∼–0.153, *p* < 0.001) and TAS-20 score (r = –0.245, 95% CI: –0.390∼–0.087, *p* = 0.003) were significantly negatively correlated with emotional module (Fig. 2). The HAMA score (r = –0.302, 95% CI: –0.442∼–0.148, *p* < 0.001) and TAS-20 score (r = –0.262, 95% CI: –0.406∼–0.105, *p* = 0.001) were significantly negatively correlated with physical module. Furthermore, negative correlations were observed among HAMA scores (r = –0.286, 95% CI: –0.427∼–0.130, *p* < 0.001), TAS-20 scores (r = –0.210, 95% CI: –0.360∼–0.051, *p* = 0.010) and other modules.

### Stepwise Regression Model Analysis

The effects of anxiety and alexithymia on QOL were discussed based on the step-by-step regression model of multiple linear regression. The ITP-PAQ scores of patients with different age, diabetes, disease stage, PLT and Hp infection subgroups were significantly different. Moreover, HAMA and TAS-20 were significantly correlated with QOL. The seven variables were defined as independent variables, and ITP-PAQ scores were set as dependent variables for stepwise regression analysis to explore the key variables affecting QOL. Among them, age and Hp infection variables were excluded because it had no significant contribution compared with the other independent variables (*p* > 0.05). The HAMA score (*p* < 0.001), the TAS-20 score (*p* = 0.015), diabetes (*p* = 0.046), disease stage (*p* = 0.027) and PLT (*p* = 0.032) all had significant effects on patients’ QOL (ITP-PAQ score, Table 5).

**Table 5.** **Stepwise regression model analysis for quality of life**.

Independent variable	Nonnormalised coefficient	Standardisation coefficient		t value	*p* value	VIF	R	R²	Adjusted R²
	B	Standard error	β						
Constant	94.90	5.43	-	17.47	<0.001	-	0.48	0.28	0.23
Anxiety	–0.71	0.21	–0.26	–3.45	<0.001	1.03			
Alexithymia	–0.22	0.09	–0.19	–2.47	0.015	1.04			
Diabetes (yes)	–4.02	2.00	–0.15	–2.02	0.046	1.03			
Disease stage (initial ITP)	–3.36	1.50	–0.17	–2.23	0.027	1.03			
PLT (≤100 × 10^9^/L)	–3.18	1.46	–0.16	–2.17	0.032	1.02			

VIF, variance inflation factor; PLT, platelet.

### Evaluation of Stepwise Regression Model

The VIF values of the six variables included in the stepwise regression model were all less than 5, indicating the lack of collinearity among the variables (Table 5). The model’s R^2^ value was 0.28, with an adjusted R^2^ of 0.23, indicating that the QOL is explained by more than one-fifth of the strength, and the model has a certain degree of fit (Table 5). In addition, the F-test was used to evaluate the overall significance of the model (Table 6). The results show that the model was significant and valid (F = 8.34, *p* < 0.001).

**Table 6.** **Significance test of stepwise regression model**.

Type	SS	df	MS	F value	*p* value
Regression	3245.59	5	648.72	8.34	<0.001
Residual	11,050.13	142	77.82		
Total	14,293.72	147			

SS, sum of squares; df, degrees of freedom; MS, mean square.

## Discussion

As an autoimmune disease, ITP threatens patients’ physical health and, due to recurrent episodes and treatment-related adverse reactions, imposes burdens their mental health and QOL [26]. This study systematically assessed how anxiety and alexithymia impact QOL in adult patients with ITP to inform clinical interventions. Our results showed high levels of anxiety and alexithymia in patients with ITP, both of which were correlated negatively with QOL. These findings highlight the complexity of the psychological status of patients with ITP and underscore the need to address mental health alongside physical symptoms in clinical management.

Firstly, this study found generally high levels of anxiety in patients with ITP, consistent with previous findings [5, 27]. The impact of anxiety, however, extends beyond the psychological distress it causes. From a pathophysiological perspective, anxiety can activate the sympathetic nervous system and the hypothalamic–pituitary–adrenal axis [28]. This activation leads to the secretion of stress hormones, such as epinephrine and cortisol [29], which can promote a pro-inflammatory state characterised by elevated levels of cytokines, such as interleukin-6 (IL-6) and tumour necrosis factor-alpha (TNF-α) [30]. In patients with ITP, whose immune system is already dysregulated, this anxiety-induced inflammatory response may exacerbate the underlying autoimmune pathology, potentially increasing platelet destruction and hindering treatment efficacy. Furthermore, the physiological burden of chronic anxiety often includes poor sleep quality, which can impair the body’s restorative functions and diminish performance in daily activities and physical health [31]. In summary, anxiety exerts a multifaceted negative impact on the QOL of patients with ITP, spanning psychological, physiological and social dimensions. Therefore, the timely identification and management of anxiety are crucial not only to alleviate psychological burden but also to improve overall patient outcomes.

The relationship between alexithymia and QOL is also noteworthy. In this study, the TAS-20 score was significantly negatively correlated with the ITP-PAQ score. A central feature of alexithymia is difficulty recognising and describing one’s own emotions [10]. Patients with ITP themselves face a variety of stresses associated with the disease, such as disease uncertainty, bleeding risk and treatment side effects. Alexithymia makes patients unable to accurately understand and express these emotions, resulting in a backlog of negative emotions in the heart [32]. The long-term accumulation of such emotions will lead to serious psychological problems such as anxiety and depression, which directly affect the psychological state and emotional stability of patients and thus reduce their QOL. Alexithymia also affects the patient’s perception of themselves and their ability to cope with the illness. Without a clear understanding of their own emotions and needs, patients have difficulty in developing effective coping strategies to deal with the problems brought about by the disease. They may not be able to realise the relationship between certain emotional changes and the disease, thereby missing the opportunity to adjust their mindset and lifestyle [33]. For example, during the remission period, patients may not be able to perceive that their inner anxiety is actually related to the fear of disease recurrence due to dysphoria. Moreover, they may not be able to take proactive measures to alleviate anxiety, which affects their recovery outcome and QOL. Meanwhile, the lack of self-awareness may make it difficult for patients to make decisions in their daily life that are appropriate for their condition, such as in diet, exercise and work arrangements, further affecting their QOL [34]. Therefore, in the clinical management of patients with ITP, scholars need to focus on psychological status, especially emotional cognition and expression, and targeted psychological interventions should be provided in a timely manner to help patients cope with the psychological stress caused by the disease.

Age, diabetes, disease stage, platelet level and Hp infection also significantly affected the QOL of patients with ITP. The ITP-PAQ scores of patients older than 50 years old were significantly lower than those of patients younger than 50 years old. With age, the body’s function declines, the tolerance to diseases is weakened and multiple chronic diseases combine, which aggravates the burden on the body and affects the QOL [35]. The lower QOL in patients with comorbid diabetes is due to the fact that diabetes itself causes multiple complications that affect the function of various body systems, which interact with ITP to exacerbate the disease and lead to limitations in physical functioning and activities of daily living [36]. The QOL of first-episode patients is lower than that of persistent or chronic patients probably because the former do not have sufficient knowledge of the disease and face a greater psychological shock when facing a sudden onset of the disease; moreover, it is often more difficult for them to adapt to the changes in their lives brought about by the disease in the early stages. The lower QOL in patients with PLT levels ≤100 × 10⁹/L may be due to the fact that low PLT levels increase the risk of bleeding, which puts the patient in a state of constant worry [37]. However, in the stepwise multiple linear regression analysis, age and Hp infection were excluded by the model. When they were combined with other variables included in the model (such as anxiety levels, alexithymia, etc.), they had relatively weak independent explanatory power for the QOL and failed to meet the statistical criteria for inclusion in the model. These results suggest that when conducting clinical treatment, we should consider the physiological and psychological factors of the patient and develop a personalised treatment plan to improve treatment outcomes and QOL.

This study has some limitations. Firstly, this single-centre study had a relatively limited sample size, which may not fully reflect the overall situation of patients with ITP. In the future, multi-centre and large sample studies should be conducted to validate the findings. Secondly, this study mainly focused on the effects of anxiety level and degree of alexithymia on QOL but did not explore the underlying neurobiological mechanisms in depth. In the future, combining neuroimaging, genetics and other research methods will contribute to an in-depth understanding of the mechanisms underlying the occurrence and development of psychological problems in patients with ITP. Furthermore, the QOL of patients is the result of the combined effect of multiple factors. The results only identified a small portion of the influencing factors (26%). Future research needs to incorporate additional potential variables. Finally, interventions were not explored, and future research could identify and develop effective interventions for anxiety and narrative disorders in patients with ITP to improve their QOL. In summary, this study reveals the significant negative impact of anxiety level and degree of alexithymia on the QOL of patients with ITP and emphasises the importance of paying attention to patients’ psychological status in clinical management. Future studies may further explore the role of psychological interventions in improving the QOL and provide a strong basis for clinical practice.

## Conclusion

This study demonstrated that adult patients with ITP exhibit high levels of anxiety and alexithymia, which are significantly and independently associated with reduced QOL. Multifactorial regression analysis identified anxiety, alexithymia, diabetes comorbidity, advanced disease stage and low PLT count as key risk factors for impaired QOL in this population. These findings highlight the critical need to integrate psychological assessment and intervention into ITP management. Specifically, routine screening for anxiety and alexithymia, coupled with targeted psychosocial support, may mitigate their detrimental effects on QOL. The results provide novel insights into modifiable psychological targets for clinical practice and support the development of integrated care models that address biomedical and psychosocial needs in ITP. Such approaches hold promise for improving holistic health outcomes in this patient group.

## Availability of Data and Materials

All experimental data included in this study can be obtained by contacting the first author (13919981335@136.com) if needed.
